# Multiple scattering and resolution effects in small-angle neutron scattering experiments calculated and corrected by the software package *MuScatt*

**DOI:** 10.1107/S1600576721009067

**Published:** 2021-10-20

**Authors:** Sebastian Jaksch, Vitaliy Pipich, Henrich Frielinghaus

**Affiliations:** a Forschungszentrum Jülich GmbH, Jülich Centre for Neutron Science JCNS-4 at Heinz Maier-Leibnitz Zentrum MLZ, Lichtenbergstrasse 1, D-85747 Garching, Germany; b Technische Universität München TUM, Heinz Maier-Leibnitz Zentrum MLZ, Lichtenbergstrasse 1, D-85747 Garching, Germany

**Keywords:** small-angle neutron scattering, SANS, corrections, multiple scattering, resolution

## Abstract

This article deals with multiple scattering effects that are important for the method of small-angle neutron scattering (SANS). It considers three channels for the coherent elastic, the incoherent elastic and the incoherent inelastic scattering processes. The first channel contains the desired information on the experiment. Its multiple scattering effects can be desmeared, as shown in the later sections of the article. The other two channels display a nearly constant background as a function of the scattering angle. The incoherent elastic scattering is treated by the theory of Chandrasekhar, allowing for multiple scattering even at large scattering angles. The transfer to a single representative thermalized wavelength by the inelastic scattering – as a simplification – is assumed to happen by a single scattering event. Once the transition to this altered wavelength has happened, further incoherent multiple scattering is considered. The first part of the paper deals with the multiple scattering effects of light water. In the later part of the article, deconvolution algorithms for multiple scattering and instrumental resolution of the elastic coherent signal as implemented in the program *MuScatt* are described. All of these considerations are interesting for both reactor-based instruments with velocity selectors and time-of-flight SANS instruments and may improve the reliability of the data treatment.

## Abstract 2

Calculations and desmearing of different multiple scattering effects for the small-angle scattering technique using the software package *MuScatt* are presented.

## 1. Introduction

The detrimental effects of resolution and multiple scattering on the small-angle scattering method have been discussed for about 60–70 years (Guinier *et al.*, 1955 ▸; Schmatz *et al.*, 1974 ▸; Glatter & Kratky, 1982 ▸). Nowadays, small-angle X-ray scattering (SAXS) instruments are highly optimized such that corrections with respect to resolution and multiple scattering are unnecessary (Pauw, 2013 ▸). The beam size is often smaller than the pixel size of the approximately 100 µm large detector pixels, and the short wavelengths and small scattering probabilities do not lead to significant multiple scattering. Of the commonly used scattering geometries, only the Bonse–Hart technique is still affected by non-negligible slit smearing, although the corrections for that are well described and the data treatment is straightforward (Pauw *et al.*, 2021 ▸; Adams *et al.*, 2019 ▸). Small-angle neutron scattering (SANS) instruments generally have more modest resolution settings than SAXS instruments for reasons of intensity (Schmatz *et al.*, 1974 ▸). For reactor-based instruments, the wavelength band often lies in the 10% range and the geometrical resolution takes similar values. The resolution problem remains at spallation sources such as the European Spallation Source (ESS) because many SANS instruments are built with short collimation and sample-to-detector distances in order to cover a wider wavelength band (Andersen *et al.*, 2020 ▸). For very small angle neutron scattering (VSANS), the typically larger structures in the micrometre size range enhance multiple scattering effects at smaller *Q* values (Pipich *et al.*, 2020 ▸). For ultra-small-angle neutron scattering (USANS), the Bonse–Hart technique is applied with considerable slit smearing (Barker *et al.*, 2005 ▸; Adams *et al.*, 2019 ▸). An introduction to SANS and SAXS can be found in recent textbooks (Hamley, 2021 ▸; Roe, 2000 ▸).

Multiple scattering effects in small-angle scattering experiments were described by the rather simple equations of Schelten & Schmatz (1980 ▸). This theory assumes that the scattering angles are all sufficiently small that the path length is ‘nearly’ equal to the sample thickness. At some scattering angles approaching 30° used in SANS, this approximation no longer holds and may introduce errors (Brûlet *et al.*, 2007 ▸).

A more elaborate theory was proposed by Chandrasekhar (2013 ▸), which allowed for longer path lengths and larger scattering angles. This theory models the sample as a slab, extending infinitely in the lateral dimension, which in experimental terms means that the sample holder must leave enough space at the sample exit. A theoretical hybrid description was introduced by Frielinghaus (2018 ▸), where coherent and incoherent scattering were split into separate contributions. The multiple small-angle scattering effects could still [as in the original theory of Schelten & Schmatz (1980 ▸)] be described by a simple equation (Frielinghaus, 2018 ▸) if the macroscopic differential cross section and the observed intensity were Fourier transformed in the two dimensions of the detector plane. For isotropic data, the 2D Fourier transform can be replaced by the Hankel transform. The advantage of this treatment is the option for removing multiple scattering effects by inverting the calculation. This approach was implemented numerically by Monkenbusch (1991 ▸) using a 2D fast Fourier transform (2D-FFT), which also allowed for non-calibrated small-angle scattering data in contrast to the original formulation (Schelten & Schmatz, 1980 ▸). An analytical approach for calculating the coherent multiple scattering effects was given by Jensen & Barker (2018 ▸). The second contribution in this hybrid treatment is the isotropic incoherent scattering, which is effectively treated in the theory of Chandrasekhar. Although Chandrasekhar’s theory could also be extended to *Q*-dependent scattering laws, it always calculates the multiple scattering effects without the option of removing them.

The first resolution corrections for SANS data were performed by smearing a model function that was then fitted to the non-desmeared experimental data (Pedersen *et al.*, 1990 ▸). In this way, the model function covered the whole *Q* range that fed into the smearing algorithm. Furthermore, it was shown that smearing sharp peaks or dips in the scattering model is more robust than desmearing the experimental data. The latter was also considered much more difficult owing to the experimental noise of the data. A realization of this is the Lake algorithm (which we access through the well known software *IRENA*), which performs the smearing of an optimized calculated scattering function to fit the desmeared experimental data (Ilavsky & Jemian, 2009 ▸; Lake, 1967 ▸). This particular method will also be briefly discussed below. An independent desmearing routine was developed by Wignall *et al.* (1988 ▸). A resolution function for time-of-flight (TOF) SANS instruments has been developed independently of that for the velocity-selector-based instruments (Mildner *et al.*, 1986 ▸; Nelson & Dewhurst, 2014 ▸; Heenan *et al.*, 1997 ▸; Heenan & Rennie, 1993 ▸). However, for TOF SANS instruments the resolution is tunable. The dynamic *Q* range is usually very high, often above *Q*_max_/*Q*_min_ ≃ 1000 (this is also true for TOF USANS; Agamalian & Koizumi, 2011 ▸; Carpenter & Agamalian, 2010 ▸).

In contrast to classical SANS experiments, the correction of USANS data has been developed to a high standard. The influence of slit collimation (and wavelength) smearing can be corrected using the algorithms of Lake and Ilavsky/Barker/Kline (Lake, 1967 ▸; Adams *et al.*, 2019 ▸; Kline, 2006 ▸). The quality of the treatment seems sufficient for the experimental data with its typical experimental noise. In USANS experiments, the investigated structures are in the micrometre size regime and thus scatter strongly. Limited acceptance of scattered neutrons reduces the effect of incoherent scattering. However, multiple scattering remains an issue.

For subtracting the incoherent background from experimental SANS data, a deeper understanding of its origins is desired. However, for reactor-based instruments with a narrow distribution of the wavelength λ, an easy subtraction of a constant background can be performed (Wignall & Bates, 1987 ▸). This becomes more complicated for TOF-based instruments. The background level becomes wavelength dependent and cross-talk between different λ slices usually occurs (Do *et al.*, 2014 ▸; Heenan *et al.*, 1997 ▸). For soft-matter research, the inelastic scattering from water and other solvents is present for most SANS experiments (Ghosh & Rennie, 1999 ▸; Sokolova *et al.*, 2019 ▸; Balacescu *et al.*, 2021 ▸) and must be subtracted properly. In addition, the usually weak *Q* dependence of the background may influence the coherent signal (Ghosh & Rennie, 1990 ▸), although this effect has never been calculated quantitatively (Barker & Mildner, 2015 ▸). The scattering from water is only well understood on much smaller length scales (Amann-Winkel *et al.*, 2016 ▸).

The general purpose of this work is to describe SANS from soft-matter samples which exhibit little or no wide-angle scattering. This is usually the case for samples containing either very small or no crystallites (Holderer *et al.*, 2020 ▸). In this paper we summarize the equations of multiple scattering on the basis of population equations. This now also includes the channel of inelastic scattering. Quantitative comparisons with water and plexiglass scattering are made. Furthermore, we provide the corrections to the elastic scattering signal, for which we developed the program *MuScatt* (Frielinghaus, 2021 ▸; Wuttke, 2021 ▸). Here, we use a 2D-FFT for isotropic data to take into account both multiple scattering and resolution desmearing. The 2D-FFT is computationally highly robust against numerical noise. Furthermore, many details of the calculations have been developed to suppress numerical noise from the 2D data spreading, from the actual correction in ‘real’ space and from the data collection for the 1D data retrieval. The result is a corrected data set with a high fidelity of the experimental noise but with clear corrections to the two different smearing effects. We present a few examples to demonstrate data correction with the *MuScatt* desmearing program. Finally, a few more thoughts on corrections for SANS data collected at spallation sources are discussed.

## 2. The scattering theory

The definitions, terminology and nomenclature used in this section refer to the original article by Frielinghaus (2018 ▸) and have been extended to include inelastic scattering from the solvent. The aim of the theory is twofold: to describe the coherent multiple scattering correctly which then allows for its removal, and to connect the incoherent background to transmission measurements at a broader wavelength range. The latter method becomes important for TOF SANS instruments in order to improve the predictive power of the background subtraction for higher wavelengths where the counting statistics are weak and the intensities at the highest scattering angles may not level off at a constant background level. A list of symbols is given in Appendix *A* [App appa]. The primary intensity is denoted by *I*_0_ and decays exponentially according to the transmission function. The elastic coherent small-angle scattering signal is *i*_1_(*z*, **Q**) and may be influenced by multiple scattering or resolution effects. The momentum transfer *Q* is connected to the scattering angle ϑ via 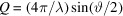 , where λ is the wavelength of the neutrons. The vector **Q** extends in the *Q*_*x*_ *Q*_*y*_ plane and is not to be confused with the **z** direction along the axis of the collimated neutrons within the sample. The coherent scattering depends on the sample thickness at *z* = *d* (slab geometry) and the scattering vector **Q**. The elastic incoherent scattering in the forward and backward directions is denoted by *j*_+_(*z*, ϑ) and *j*_−_(*z*, ϑ). Additionally, we treat the inelastic scattering which we model as intrinsically *Q* independent – similar to the incoherent contribution – in the forward and backward directions: *k*_+_(*z*, ϑ) and *k*_−_(*z*, ϑ). The overall integro-differential equations then become

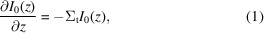




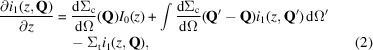




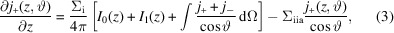




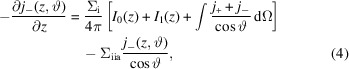




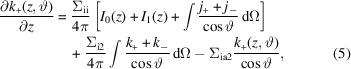




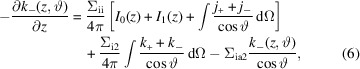

with

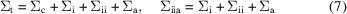

and

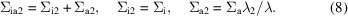

We now have the scattering probabilities of the coherent, the incoherent and the incoherent inelastic scattering and the absorption given by Σ_c_, Σ_i_, Σ_ii_ and Σ_a_, respectively (the integral cross section Σ_c_ results from the macroscopic differential cross section dΣ_c_/dΩ by integrating over all solid angles). The idea behind the inelastic scattering contribution is that the final wavelength is a thermalized wavelength that is determined by the Maxwell distribution and takes the average value of λ_2_ = 1.8 Å at 298 K or 0.025 eV (averaged over the energy). The final incoherent wavelength is independent of the incoming wavelength λ and is assumed to be achieved by a single scattering event (Ritenour *et al.*, 1990 ▸). This assumption is never completely true (Ghosh & Rennie, 1990 ▸; Heenan *et al.*, 1997 ▸; Heenan & Rennie, 1993 ▸). We assume that the neutron takes either the original wavelength λ or the fixed wavelength λ_2_ and no other wavelengths are allowed. This is an idealization of the otherwise broader distributions, especially of the inelastically scattered neutrons. The further inelastic scattering and absorption probabilities Σ_i2_ and Σ_a2_ scale with the wavelength.

The idea behind equations (1)–(6) [Disp-formula fd3] [Disp-formula fd4] [Disp-formula fd5] [Disp-formula fd6] is that of population equations. On the left-hand side the differentials indicate the changes to single channels of intensity that are determined by specific scattering probabilities on the right-hand side. A scattering probability consists of a macroscopic differential or total cross section and an intensity channel from where or to where (+ and − sign) the neutrons transition. Also, the intensity channel in this product can be a specific intensity to a certain scattering angle (lower-case letters) or an integral intensity (upper-case letters). The specific intensities to a certain scattering angle always carry the reciprocal unit of the solid angle to which the intensity is scattered. The overall unit of this product (per solid angle or integral) is connected to the unit of the left-hand expression. The products may also contain integrals, which means that all specific scattering angles (in the respective hemisphere) of the intensity channels need to be considered.

The respective integrals of the multiple scattering problems are all two dimensional. Later we will introduce the Fourier transform of the coherent scattering *i*_1_ and the macroscopic differential cross section dΣ_c_/dΩ in the *xy* plane. This will lead to a factorization of the convolution in equation (2). For the incoherent scattering channels, however, the integrals cannot be calculated explicitly and the respective equations remain integro-differential equations.

There are some assumptions connected to this description. The multiple coherent small-angle scattering takes place at small angles where the variation of the respective sample thickness does not matter. As we will see later, the real shape changes of the scattering curves happen at relatively small scattering angles, while an overall calibration factor may shift the whole curve to higher intensities. Also, the wide-angle scattering can safely be neglected for most soft-matter samples containing either no or very small crystalline domains (Holderer *et al.*, 2020 ▸). The description assumes that there is no impediment to the flight of incoherently scattered neutrons exiting the sample at large angles. This also includes flight paths of neutrons that are first scattered in a lateral direction, travel a considerable distance within the sample and are then scattered towards the detector. So, laterally a larger exit window of 1–2 mm to either direction in the *xy* plane would be beneficial. Of course this is not always the case, and these kinds of extreme flight paths have been examined in detail elsewhere (Carsughi *et al.*, 2000 ▸). The third assumption is that neutrons being scattered incoherently once do not contribute further to the coherent channel. This means that the information about the scattering angle is lost by the first incoherent scattering event, and secondly that the widening by small-angle scattering is not relevant to the incoherent channels. The same assumption applies to the neutrons being scattered once inelastically. They will then keep their thermalized wavelength.

**Figure 1:**
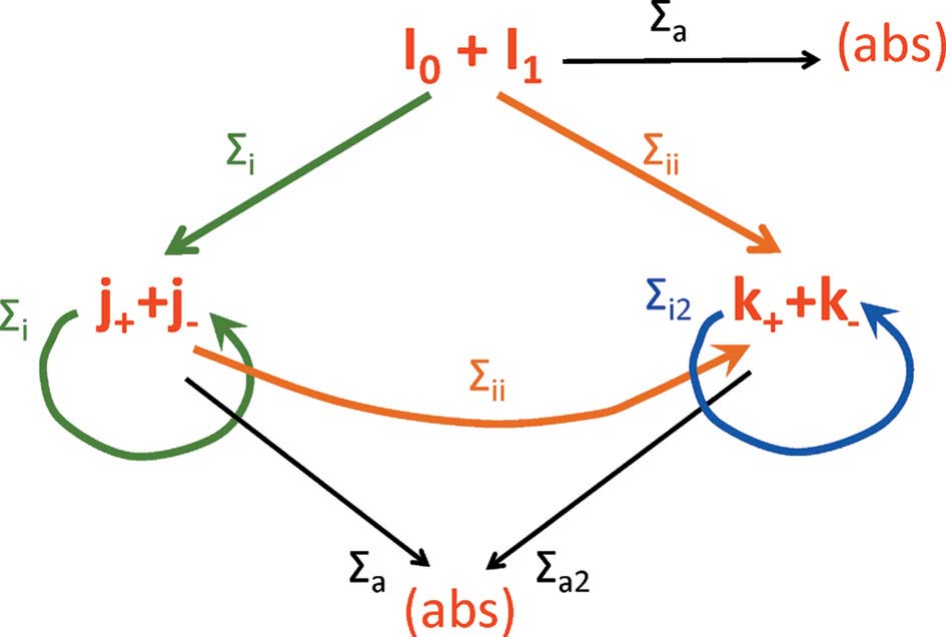
Simplified graphical explanation of the population equations (1)–(6) [Disp-formula fd3] [Disp-formula fd4] [Disp-formula fd5] [Disp-formula fd6]. The three populations (*I*_0_ + *I*_1_), (*j*_+_ + *j*_−_) and (*k*_+_ + *k*_−_) arising from the intensity at small angles, the elastic incoherent intensity and the inelastic incoherent intensity are considered. Arrows represent the possible scattering routes. Accordingly, there are arrows from the small-angle intensity towards the other two intensities with probabilities Σ_i_ and Σ_ii_. Multiple scattering contributions are represented by circular arrows. Importantly, there are two routes to the inelastic intensity with the probability Σ_ii_ that amplify its population considerably. More details about the separate populations are given by Frielinghaus (2018 ▸).

Frielinghaus (2018 ▸) provided a graphical representation of the different transitions of the neutrons between the different populations *I*_0_, *i*_1_, *j*_+_ and *j*_−_. The idea behind this idealization is that the small-angle scattering takes place at small angles where the path length is not changed, but comes into play via the 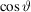 terms in the incoherent scattering. We call the integral coherent scattering population 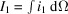 . The new transition via the inelastic scattering is a new route to the populations *k*_+_ and *k*_−_. Once again this is a one-way route: after the neutron takes the wavelength λ_2_, there is no way back to the other populations. We now discuss the three populations (*I*_0_ + *I*_1_), (*j*_+_ + *j*_−_) and (*k*_+_ + *k*_−_) as depicted in Fig. 1 ▸. There is always a loss due to absorption, but from the first population (*I*_0_ + *I*_1_) there are two important possibilities of either elastic incoherent or inelastic incoherent scattering. From the two populations (*j*_+_ + *j*_−_) and (*k*_+_ + *k*_−_) there are multiple scattering routes that redistribute the intensities with respect to the angle ϑ. Lastly, there is another transition from (*j*_+_ + *j*_−_) to (*k*_+_ + *k*_−_) via the inelastic scattering probability. Note that the inelastic population has two sources: the coherent and incoherent elastic populations. The additional pathway contributes significantly to the growth of the inelastic population and therefore both pathways must be taken into account.

The different scattering contributions can be solved sequentially. For the primary intensity we obtain 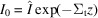 with 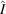 being the full primary intensity entering the sample. The total coherent scattering is described by 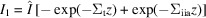 . It has a maximum at 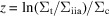 and the ideal sample thickness *d* is chosen accordingly in order to optimize the macroscopic differential cross section in the scattering experiment.

The multiple coherent scattering solution is obtained by using the Fourier transform. Any function in **Q** space will be transformed to real **r** space via 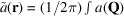 × 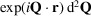 . The back-transform is done by the expression 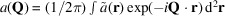 . The treatment of the coherent multiple scattering problem only introduces the integrals over the detector plane and does not involve the **z** direction. The underlying mathematics only considers a surface and not a volume. At this point, the formalism represents a complete and correct description of anisotropic scattering. For isotropic scattering, the Fourier transform can be carried out in one dimension according to 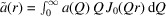 and for the back-transform according to 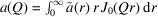 , where *J*_0_ is the Bessel function of zero order. These are the Hankel transforms of zeroth order. At any point, one may return to anisotropic scattering by including the full vectorial dependence on **r**. The already well known (Schelten & Schmatz, 1980 ▸; Monkenbusch, 1991 ▸) analytical solution for the elastic coherent intensity 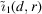 (in ‘real’ space) then reads

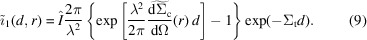

Note that for small scattering signals the single scattering solution is obtained asymptotically [ 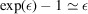 ]. The difficulty of this equation is the separation of the coherent and overall incoherent scattering [in terms of Σ_c_ and Σ_i_ + Σ_ii_ or *i*_1_ and (*j*_+_ + *k*_+_), which will be observed as a sum on the detector]. Examples of dealing with multiple coherent scattering events are discussed below in Section 6.

The elastic incoherent scattering intensities are described quantitatively by the theory of Chandrasekhar (2013 ▸). In summary, one obtains the following result: 

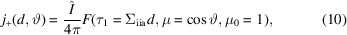




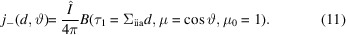

The characteristic functions *F* and *B* for the scattering in transmission and the back-scattering (also called *T* and *S* in the literature) are defined by the following equations: 

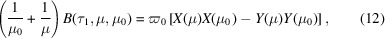




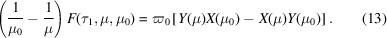

The cosine of the incident angle μ_0_ takes the value 1 for orthogonal incidence. The albedo ϖ_0_ takes the ratio Σ_i_/Σ_iia_. The details of the *X* and *Y* functions are described in the book of Chandrasekhar, and are widely discussed in the literature (Caldwell, 1982 ▸; Viik, 1986 ▸, 2021 ▸). These two functions help to reduce the original integro-differential equations (3) and (4) to integral equations. Replacing the integrals by discrete sums, the solution can then be obtained from a linear algebraic equation. For small optical thicknesses τ_1_, the classical single scattering results are obtained, *i.e.* a rather flat signal that directly scales with the scattering probability Σ_i_. However, the effect of large path lengths towards large angles ϑ → π/2 is always included in the description.

The solutions for the inelastic incoherent scattering are more complicated owing to the inhomogeneity of the differential equations, namely 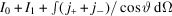 . The first two terms together take the form of a simple exponential function. The last term can be approximately described by two exponential functions and a constant, *i.e.* 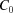 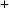 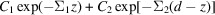 , that we fit to the intensity distribution of 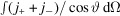 within the slab. The solution of the inelastic scattering is linear with respect to the inhomogeneity, and therefore the four solutions emerging from the four single terms of the inhomogeneity are fully independent. Usually, the apparent probabilities determined from the inhomogeneity (*j*_+_ + *j*_−_), for instance Σ_1_, are larger than the physically relevant values encountered in the scattering problem, *i.e.* Σ_ia2_. Thus, the decay of the inhomogeneity is faster than one would expect from the original physical problem. This can be simulated by using a different incident angle for a hypothetical incoming radiation, *i.e.* μ_0_ = Σ_ia2_/Σ_1_. Thus, the solution of the inelastic scattering is calculated in a similar fashion to equations (10)–(13) [Disp-formula fd12] [Disp-formula fd13], in this case with four different summation terms. If the single decays of the four inhomogeneities are too slow, further approximations are necessary. As for the simplest case, we then approximate this slow decay by a superposition of a constant and a decay with Σ_ia2_. However, this is only required for extremely high (∼19 Å, which should be avoided – see kink in Fig. 3) neutron wavelengths in combination with strong incoherent scatterers like H_2_O. As this combination of very high wavelengths and aqueous solvent is rarely used, we did not implement the full correction in this work. Note that the prefactors for the different contributions now read 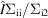 , 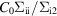 *etc*. The albedo here is ϖ_0_ = Σ_i2_/Σ_ia2_.

## 3. Water and plexiglass scattering

**Figure 2:**
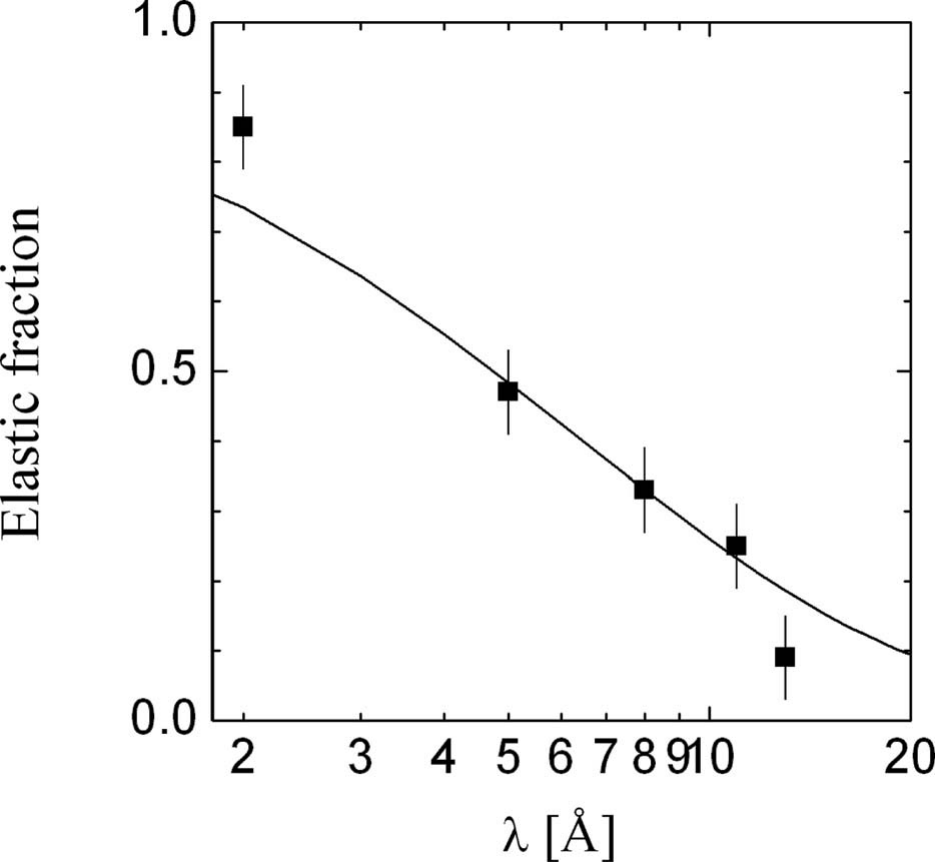
The elastic fraction of the small-angle neutron scattering of a 1 mm thick water sample as a function of wavelength (Ghosh & Rennie, 1999 ▸). The solid line represents the simulation carried out on the basis of equations (1)–(6) [Disp-formula fd3] [Disp-formula fd4] [Disp-formula fd5] [Disp-formula fd6].

One basis for describing water scattering is the publication by Ghosh & Rennie (1999 ▸). Here, the SANS spectra of water (and other samples) with a sample thickness of 1 mm were measured for different incident wavelengths and the ratios of the elastic versus the total (elastic and inelastic) scattering were determined. The detector-efficiency-corrected elastic fractions are displayed in Fig. 2 ▸. For the solutions of equations (1)–(6) [Disp-formula fd3] [Disp-formula fd4] [Disp-formula fd5] [Disp-formula fd6], the incoherent scattering cross section and the absorption cross section of water, Σ_i_ and Σ_a_, were determined using the NIST web portal (National Institute of Standards and Technology, 2021 ▸). The final wavelength of the thermalized neutrons λ_2_ was set to 1.8 Å (the average of the Maxwell distribution at 298 K or 0.025 eV; Sokolova *et al.*, 2019 ▸), and then all cross sections at that wavelength result from the wavelength scaling. The inelastic cross section was determined by adjusting the simulations of the elastic fraction for the 8 Å value to the experimental value. The nearest-neighbor data points agree well with the simulation. The values for 2 and 13 Å, however, deviate from the simulation results. This could be due to the overlap of the incoming wavelength distribution and the Maxwell distribution, and in the case of the 13 Å measurement the statistics of the measurement are considerably worse owing to the low intensity. For the simulations, we assumed a scaling of the inelastic cross section of Σ_ii_ ∝ λ. Of course, one could assume a scaling with a different exponent. However, as long as the integral scattering probabilities of both wavelengths (*i.e.* the incoming wavelength and 1.8 Å) cover the same physical features and are not limited by the available exit angles, our choice of exponent is supported by the *k*/*k*_0_ term as given by Farhi *et al.* (2015 ▸). This assumption is also supported by the fact that the monochromatic collision kernel for low incoming energies (Ritenour *et al.*, 1990 ▸) is effectively governed by a single scattering event.

**Figure 3:**
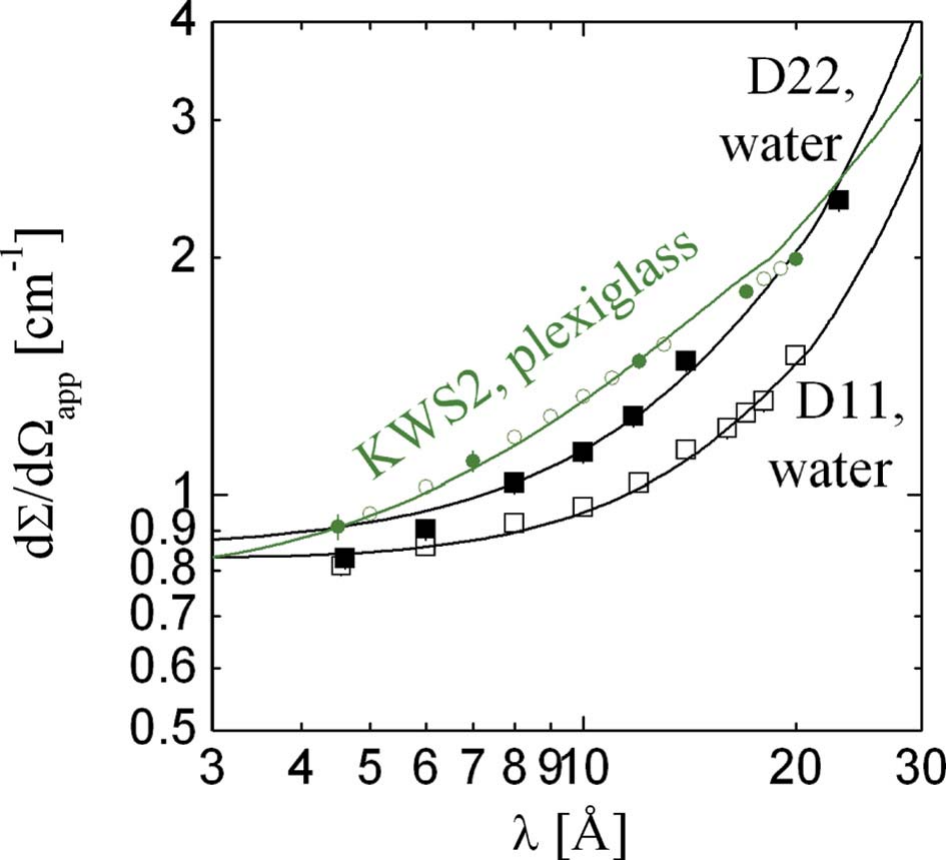
The calibrated apparent small-angle scattering macroscopic differential cross sections of water (1 mm thickness) and plexiglass (1.5 mm), measured and simulated. The black data points arise from water measurements on the instruments D11 and D22 at the Institut Laue–Langevin (ILL) (Lindner, 2000 ▸). The solid green data points arise from plexiglass measurements on the instrument KWS2 at FRM2 (internal communication). The open dots are interpolated for calibration use. The error bars are either clearly indicated or similar to the symbol size. The solid lines are simulated using equations (1)–(6) [Disp-formula fd3] [Disp-formula fd4] [Disp-formula fd5] [Disp-formula fd6].

After calibrating the inelastic scattering probability to the elastic scattering fractions, we compare the calibrated apparent macroscopic differential small-angle scattering cross sections 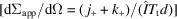 of water (Lindner, 2000 ▸) and plexiglass (a commercial polymer that in its pure form would also be called poly methylmethacrylate) with simulations [equations (1)–(6) [Disp-formula fd3] [Disp-formula fd4] [Disp-formula fd5] [Disp-formula fd6]] (Fig. 3 ▸). For the water measurements on the two instruments D11 and D22, the parameters for the relative detection efficiency of the 1.8 Å neutrons were taken as 15 and 25% [BF_3_ and ^3^He detectors; more details are given by Lindner (2000 ▸)] with respect to the higher wavelengths. For the plexiglass measurements on KWS2, the inelastic cross section Σ_ii_ was determined from the λ dependence of the transmission measurements, while the incoherent cross section Σ_i_ was calculated using the NIST scattering length density calculator (National Institute of Standards and Technology, 2021 ▸). This method becomes important for TOF SANS instruments. The relative detector efficiency for the 1.8 Å neutrons was assumed to be 62% (Li scintillation detector). The simulations for the different instruments and the two samples compare well with the experiments. We stress that only the inelastic scattering probability and the relative detector efficiency were free parameters of this description. In particular, the detector efficiency explains the considerable differences of water scattering between the two instruments D11 and D22. The most crude assumption of these simulations is that the inelastic scattering results in a single wavelength of 1.8 Å after the scattering.

**Figure 4:**
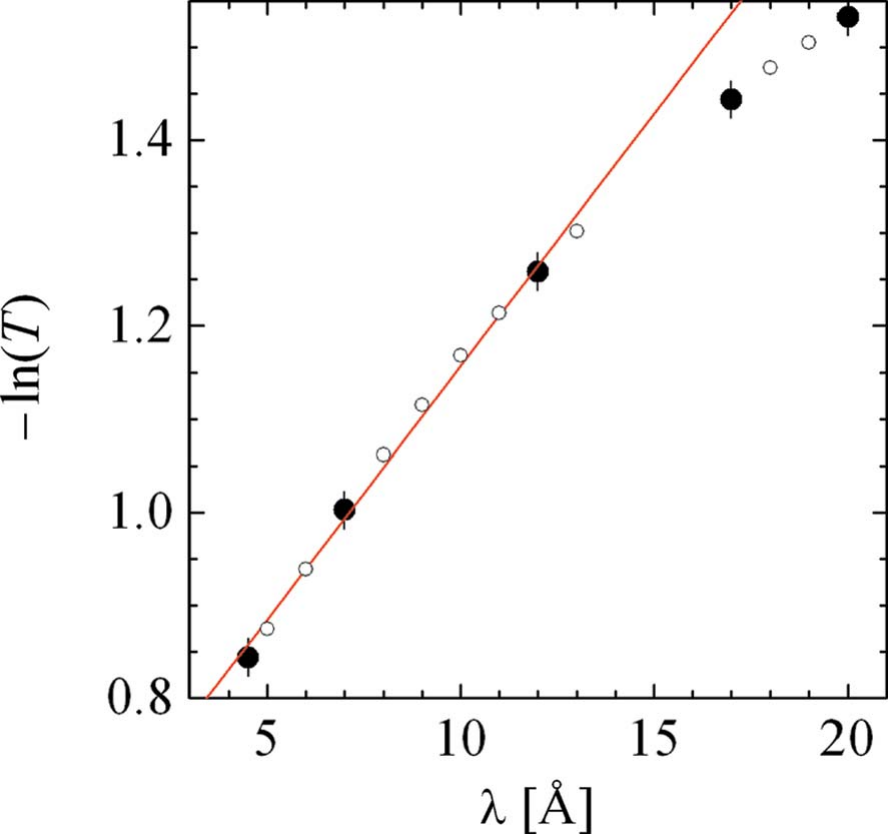
The negative logarithm of the measured (internal communication) transmission of plexiglass (1.5 mm) on KWS2 (solid points) as a function of the incident wavelength. The open symbols indicate interpolated data. The red line is a linear fit to wavelengths below 15 Å.

The transmission measurements of the same plexiglass sample on KWS2 are displayed in Fig. 4 ▸. The optical thickness is quite linear, indicating two strong contributions to Σ_t_: Σ_i_ as a constant and (Σ_ii_ + Σ_a_) ∝ λ. The latter contribution is usually dominated by the inelastic incoherent scattering for soft-matter samples. From the linear fit, we took the inelastic scattering probability Σ_ii_ and replaced the fitted elastic contribution Σ_i_ by a calculated value from the NIST web page (National Institute of Standards and Technology, 2021 ▸). These two parameters were then used to simulate the apparent macroscopic differential cross sections in Fig. 3 ▸. For samples that undergo small-angle scattering, the coherent contribution is also dependent on the square of the wavelength, Σ_c_ ∝ λ^2^. For TOF SANS instruments, this behavior is very important for the reduction of the raw data. One should therefore always compare the calibrated integral coherent scattering and the calibrated incoherent scattering background with the transmission measurements as a function of wavelength on the basis of the scattering probabilities, as Σ_c_ and Σ_ii_ scale considerably with λ.

The deviation of experimental transmissions from our linear fit (Fig. 4 ▸) at wavelengths larger than 17 Å is a matter of the physics of the long wavelengths versus shorter wavelengths. The integral scattering probabilities at large wavelengths no longer cover the same short-range structure and dynamics of the hydrogen atoms. This is also reflected in the deviation of the measured plexiglass apparent macroscopic cross section (Fig. 3 ▸).

**Figure 5:**
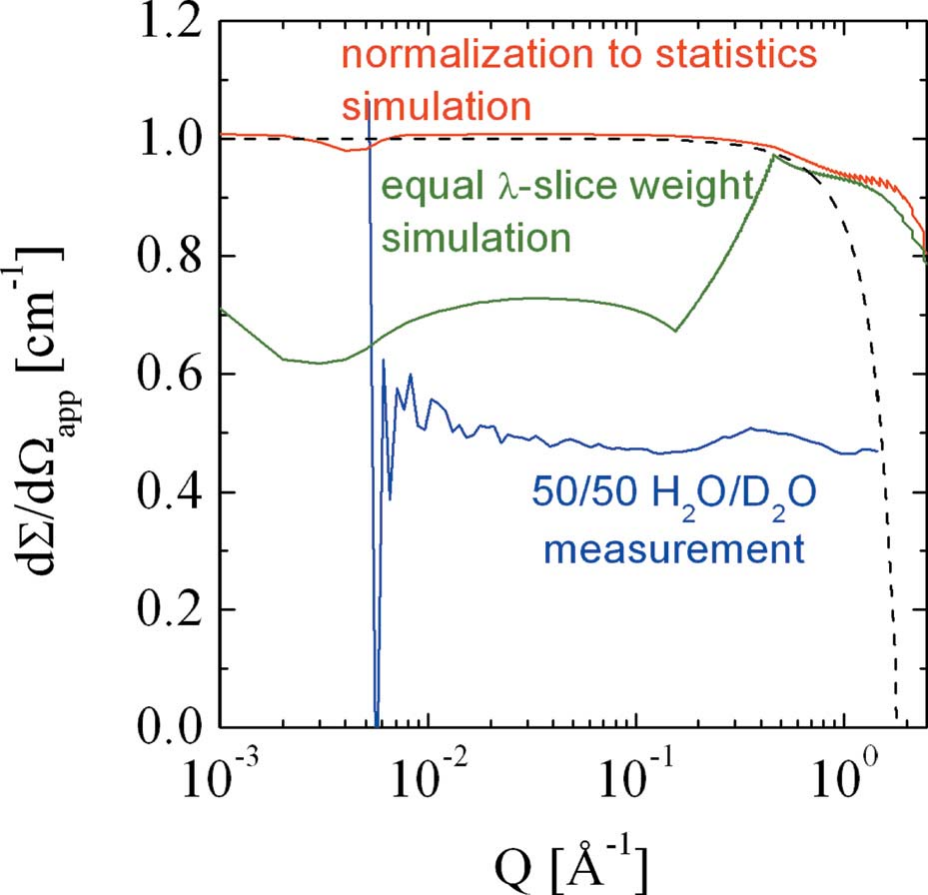
The water scattering simulated as a function of the scattering vector magnitude *Q*. The dashed black curve displays the water scattering for a reactor-based instrument at 5 Å wavelength. The red curve shows simulated water scattering for the ISIS instrument SANS2d, with statistical weight from the intensity of the spectrum of a cold source. The green curve shows a simulation of the same instrument with equal weights for each λ slice. The fringes at high *Q* result from the distinct binning of λ slices at intervals of 0.25 Å. The blue curve displays experimental data from a 50:50 mixture of H_2_O/D_2_O measured on SANS2d.

The angle-dependent water scattering is shown in Fig. 5 ▸ as a function of the scattering vector magnitude *Q*. The dashed black line indicates the water scattering for a reactor-based SANS instrument (for instance KWS2) with a wavelength of 5 Å. The steep drop-off in intensity lies in the wide-angle range – far beyond the classical SANS *Q* range. The important result is that the background is *Q* dependent, even in the typical SANS range of approximately 0.001–1 Å^−1^. Thus, it may affect the coherent scattering when determining the radius of gyration from a polymer solution.

So far, we have simulated only a single incoming wavelength and neglected the distribution arising from the velocity selector. However, for time-of-flight SANS instruments the use of many different wavelengths (1.5–12 Å) is necessary by design. To address this issue, we superimposed a range of wavelengths with a 0.25 Å step in a pulse that is used by the instrument, and took the different speeds of the inelastic scattered neutrons of 1.8 Å into account. We accounted for the Maxwell distribution of intensities from a typical cold source (with an additional factor λ^−1^ as is usually observed for most spallation sources because of window absorption effects). The collected neutrons were then calibrated using the transmission and sample thickness (what we call apparent calibration). The different slices were weighted either equally (green curve) or statistically by the intensity of the incoming neutrons (red curve). The geometrical dimensions of the instrument SANS2d (Heenan *et al.*, 2011 ▸, 2006 ▸) were considered for this simulation, with a wavelength band from 1.5 to 12 Å. The fringes that occur at high *Q* are an artifact of the wavelength binning and we do not consider them to invalidate the method. The green curve, though, is far from flat and can be considered a poor representation. However, the interesting effect lies in the maximum at *Q* = 0.4 Å^−1^, which could develop into a water peak as observed for an H_2_O/D_2_O mixture measured on SANS2d (Heenan *et al.*, 2011 ▸) (blue curve, personal communication) using different (incorrect) statistical weights. The different wings of the maximum at *Q* = 0.4 Å^−1^ (green curve) originate from two effects: From the left, the weakly contributing λ slices give way to strongly contributing slices, leading to an increase in intensity towards higher *Q*. To the right, the inelastic contributions decay towards lower wavelengths. To rephrase this issue of the inelastic scattering intensity: the Maxwell distribution of the cold source with respect to the unaltered, original wavelength for the inelastic pathway is often much lower than that for the elastic incoherent intensity. Both terms are equally important only close to the maximum of the Maxwell distribution of the cold source. Overlap between different pulses must be avoided for two reasons: to avoid the overlap of two time frames within the assumption of elastic scattering only, and to avoid the overlap from inelastic scattering of the lowest wavelengths that hits the detector even earlier if the lowest wavelengths are larger than 1.8 Å.

The optimum operation of a time-of-flight SANS instrument at the ESS should include a careful transmission measurement that is efficiency corrected for the wavelength and should take place within the primary beam using a strongly collimated beam. As we know, this is naturally performed at pulsed sources. From the wavelength dependence of the transmission measurements – as measured – one could separate the different contributions of the scattering probabilities: elastic incoherent scattering (wavelength independent), inelastic incoherent scattering (and absorption ∝ λ) and possibly the coherent elastic SANS scattering (∝ λ^2^) by fitting a polynomial of second order. The transmission measurements must be performed very carefully in order to separate the bare sample transmission from the sample holder transmission. The cross sections obtained from the fitting must be compared with the experimental *Q*-dependent incoherent contributions and with the integral elastic SANS scattering. By correctly balancing the transmission and the scattering dependencies, one might obtain a higher reliability of the calibration of all scattering contributions – most importantly the coherent SANS signal. This requires a high reliability of all detection efficiencies of all detectors and monitors. For all the corrections, we have assumed typical soft-matter samples that show little or no wide-angle scattering from crystallites. In pioneering work, the separation of coherent and incoherent scattering has been addressed already (Seeger & Hjelm, 1991 ▸; Heenan *et al.*, 1997 ▸). In the case of very large scattering angles (which are now achievable at many modern SANS instruments), additional corrections are required to account for the increased neutron path length within the sample. Such corrections are described elsewhere (Brûlet *et al.*, 2007 ▸).

## 4. Deconvolution of experimental data

So far, we have considered only the theoretical calculation of the scattering signals – mainly of water scattering. The whole theory also allows us to deconvolute the multiple scattering effects that occur in elastic SANS scattering. Furthermore, it is useful to consider the instrumental resolution effects for experimental data. The description of these effects requires a two-dimensional Fourier transform of the experimental data set. We use a 2D-FFT code from Ooura (2021 ▸). The corrections of the scattering profiles are performed in real space – also known as *r* space. [The real space that applied in a three-dimensional FFT would yield *p*(*r*), the real-space correlation function. However the multiple scattering treatment demands a two-dimensional FFT.] The data are then transformed back and can be treated further using model fitting of the scattering curves. The exact definitions for the Fourier transform are given in the text close to equation (9). We recall that the functions in different spaces are denoted 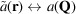 . The discrete Fourier transform is highly stable with respect to numerical noise when applied twice. Within machine precision, all data can be recovered. Much care has been taken over the distribution of the one-dimensional data on the 2D lattice and their recovery by a careful interpolation method before and after the 2D-FFT. In this way, an overall high fidelity of the corrections was achieved.

The following real-space calculations are those originally reported by Schelten & Schmatz (1980 ▸). From the simple differential equations of the multiple scattering problem (Frielinghaus, 2018 ▸) the true macroscopic differential cross section dΣ/dΩ is connected to the measured intensity *i*_1_ via 

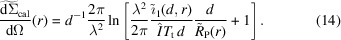

The fraction 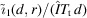 refers to the calibrated apparent scattering intensity. The primary intensity 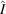 is usually measured indirectly by a secondary calibration standard, and the functional form of 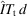 is replaced by the real calibration method. If the absolute calibration of the experiment is performed correctly, the calibrated data can be used directly for the correct correction of multiple scattering effects. The multiple scattering correction occurs in the expression 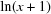 , and the Taylor expansion powers correspond to the number of scattering processes considered. The factors 2π/λ^2^ and *d* arise from the integration and intensity calculations, respectively, and serve to maintain the correct dimensionality. The function 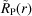 introduces a possible correction for the instrumental resolution. It will be discussed below in more detail. Equation (14) is referred to as correction method 1. A second method of correction was introduced by Monkenbusch (1991 ▸): 

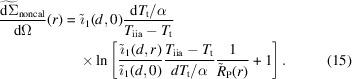

This method does not use the absolute scale of the experimental intensity but requires the transmissions 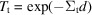 and 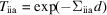 to be known accurately. *T*_t_ is the overall experimental transmission required for calibration, and *T*_iia_ is calculated on the basis of incoherent scattering and absorption. For neutrons, the partial cross sections of incoherent scattering and absorption can be calculated accurately (National Institute of Standards and Technology, 2021 ▸) and the inelastic scattering probability must be obtained from λ-dependent transmission measurements. The final result of this correction stays at the same undefined level of calibration. The numerical value of α = 0.057 cm was determined by comparison of experimental data with the three different correction methods (*i.e.* for one specific microemulsion). This factor describes the scaling of the normalized intensities 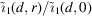 , *i.e.* the strength of multiple scattering with respect to this magnitude. Therefore, if the experimental intensity is calibrated well, the whole correction works even on an absolute scale. Equation (15) is referred to as correction method 3. A hybrid method, method 2, is obtained when the original prefactor that ensures the calibration 2π/(λ^2^ *d*) of the final result is kept: 

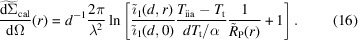

All methods 1, 2 and 3 can be reverted to add multiple scattering effects by replacing the functional form 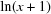 by 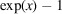 . Those corresponding methods we number 4, 5 and 6. Note that methods 1–3 remove multiple scattering effects and methods 4–6 add multiple scattering effects to the data set. All multiple scattering calculations are quite noise tolerant and we do not need to suppress high-frequency noise additionally.

In the following, we focus on resolution effects to be corrected, *i.e.* to be desmeared. The simplest resolution function is a Gaussian, where 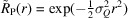 , performed individually for each scattering vector magnitude *Q*. One basic argument for this simplicity is the central limit theorem. The more contributions we include in the effective 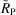 , the more the result becomes Gaussian. This idea was also taken into consideration by Pedersen *et al.* (1990 ▸), who only considered the distortion of the smearing in polar coordinates (the two-dimensional σ_*Q*_ environments – represented by small circles of radius σ_*Q*_, centered along the perimeter of a circle of constant *Q* – appear to include more contributions from slightly smaller *Q*). A further potential shortcoming of the Gaussian formula is that the presence of distribution tails at large *r* values leads to large corrections. While the essential correction is performed at small *r*, the experimental noise is simply amplified at large *r* (non-Gaussian corrections, for instance a simple slit correction, would even result in divergences in the application). We therefore require a cut-off in *r* space. This idea is represented best with the formula 

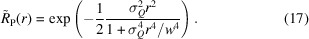

The whole formula makes a smooth cut-off, and at large *r* the function 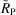 approaches unity, which preserves the high-frequency noise. The parameter *w* controls the cut-off in terms of the argument σ_*Q*_ *r*, *i.e.* at which relative *r* parameter the cut-off of the noise takes place. Applying this formula to experimental SANS data, it was found that a value of *w* = 2.0 was appropriate in most cases. The cut-off value can be tuned according to the requirements of the data, but the value should lie between 1.5 and 2.5. So, applying the resolution deconvolution in real *r* space requires a cut-off term, and any given smearing function is reduced to a simple standard deviation σ_*Q*_.

## 5. The program *MuScatt*

The program *MuScatt* is published in a GitLab repository (Frielinghaus, 2021 ▸; Wuttke, 2021 ▸). Since it is still under construction, a few more routines will be added over time. However, the core components of the program already deliver very good performance.

The kernel routine of *MuScatt* is a two-dimensional fast Fourier (cosine) transform adapted from the work of Ooura (2021 ▸). One property of this routine is that, when applying the transform twice, the original data set is recovered up to machine precision. This property is important in the sense that large and small intensities (for instance in a Porod power law) are treated together, such that small intensities may suffer from reduced precision when summed together with large intensities.

Furthermore, we restricted ourselves to isotropic SAS data that we need to spread over a two-dimensional lattice. For this purpose, interpolations must be made. This usually leads to an averaging of neighboring data points, which will occur a second time in the collection of the data from the lattice. To counteract this effect, we also check for a significant consistent change of the curvature from the neighboring data points in the original data set and apply this curvature to the interpolation. This method captures certain exaggerated features that will be averaged later in the data collection from the lattice. A linear interpolation is only applied if the curvature is not statistically significant. This detail enhanced the quality and crispness of the recovered data set.

The last important contribution to the high fidelity of the program *MuScatt* is the preservation of the experimental noise without spurious amplification. The multiple scattering corrections deal with small corrections when the argument *x* of the 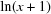 term is small. In this case, the expression was expanded as a Taylor series for small arguments *x* to display the highest degree of precision. The resolution correction comprises division by the resolution function 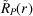 . At the high-*r* end, the cut-off parameter *w* truncates the function to unity. This ensures that higher-frequency noise is ignored and transferred unchanged to the corrected data set. Although possible, truncation effects in limited *Q* space do not usually appear, in particular because the intensities fade at high *Q* and are also associated with large errors. Figs. 11 and 12 below[Sec sec7] show how the truncation effects affect theoretical calculations. A few more details of the program are discussed in the ‘readme’ documents on the GitLab repository.

The program *MuScatt* works with isotropic ASCII data in three-column format in the following order: the scattering vector magnitude *Q* (Å^−1^), the calibrated intensity (cm^−1^) and the statistical error of the intensity (cm^−1^). In addition, *MuScatt* requires a parameter file containing the name of the ASCII scattering file, a switch for the extrapolation towards low *Q* (to determine how the beam stop data will be extrapolated), a switch for the incoherent scattering subtraction, the experimental wavelength λ (Å) and the sample thickness (cm). These are the essential parameters needed for deconvolution method 1 (introduced above). Further parameters are the experimental transmission and the calculated Σ_ia_ for the incoherent scattering and absorption, both important for methods 2 and 3. There are further switches and parameters that are explained in the readme.pdf file.

## 6. Examples for desmearing multiple scattering effects

**Figure 6:**
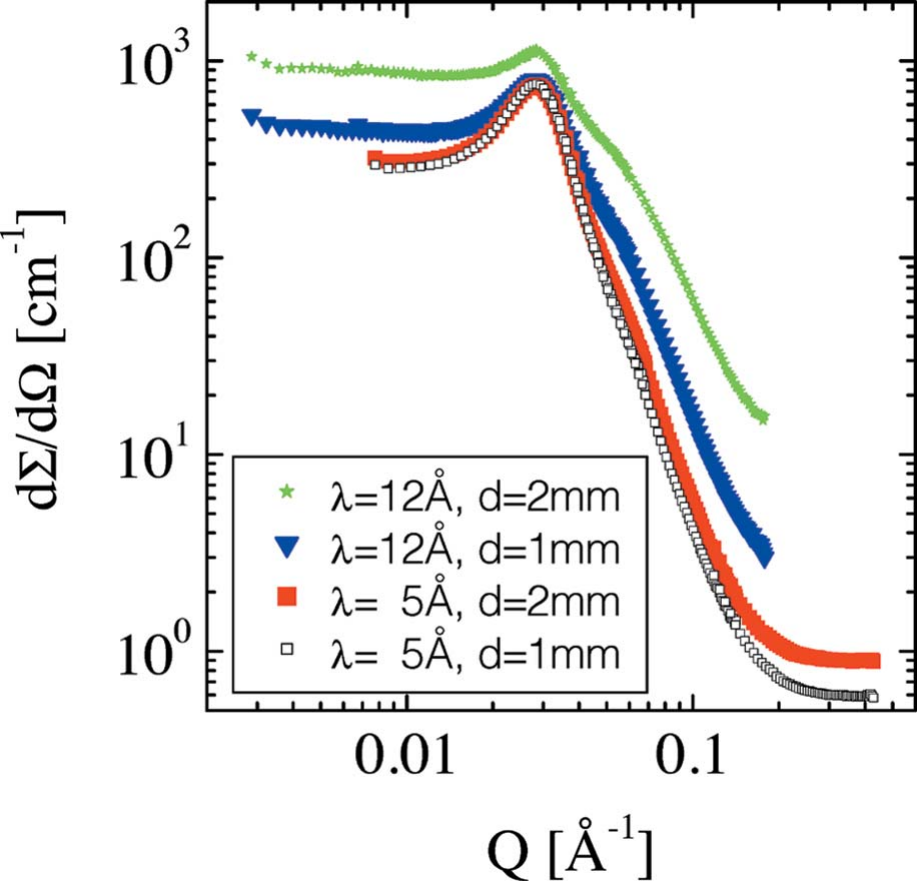
Four scattering experiments with the same microemulsion (Frielinghaus, 2018 ▸) are depicted on a log–log scale: apparent macroscopic differential cross section as a function of the scattering vector magnitude *Q*. The conditions for the neutron wavelength λ and the sample thickness *d* are indicated in the legend.

In this section, we present and discuss some example calculations to demonstrate the quality of the *MuScatt* routines. One original experimental data set has already been presented by Frielinghaus (2018 ▸). Strongly scattering microemulsions display strong multiple scattering effects when using longer neutron wavelengths or thicker samples. The data set is displayed in Fig. 6 ▸ for λ = 5 Å, *d* = 1 mm; λ = 5 Å, *d* = 2 mm; λ = 12 Å, *d* = 1 mm; and λ = 12 Å, *d* = 2 mm (λ being the neutron wavelength and *d* the sample thickness). The transmissions of the sample were *T* = 0.554, 0.307, 0.179 and 0.031, respectively, for each experimental configuration. The last data set suffers most from the presence of multiple scattering. The apparent calibration elevates the data set to higher intensities. In addition, a shoulder develops at *Q* = 0.06 Å^−1^. This is due to the double scattering of neutrons from the correlation peak at *Q* = 0.03 Å^−1^. Furthermore, the correlation peak smears out towards small *Q* and the plateau towards small *Q* is elevated. This feature arises as a result of the low specificity of the multiple scattering in terms of *Q*.

**Figure 7:**
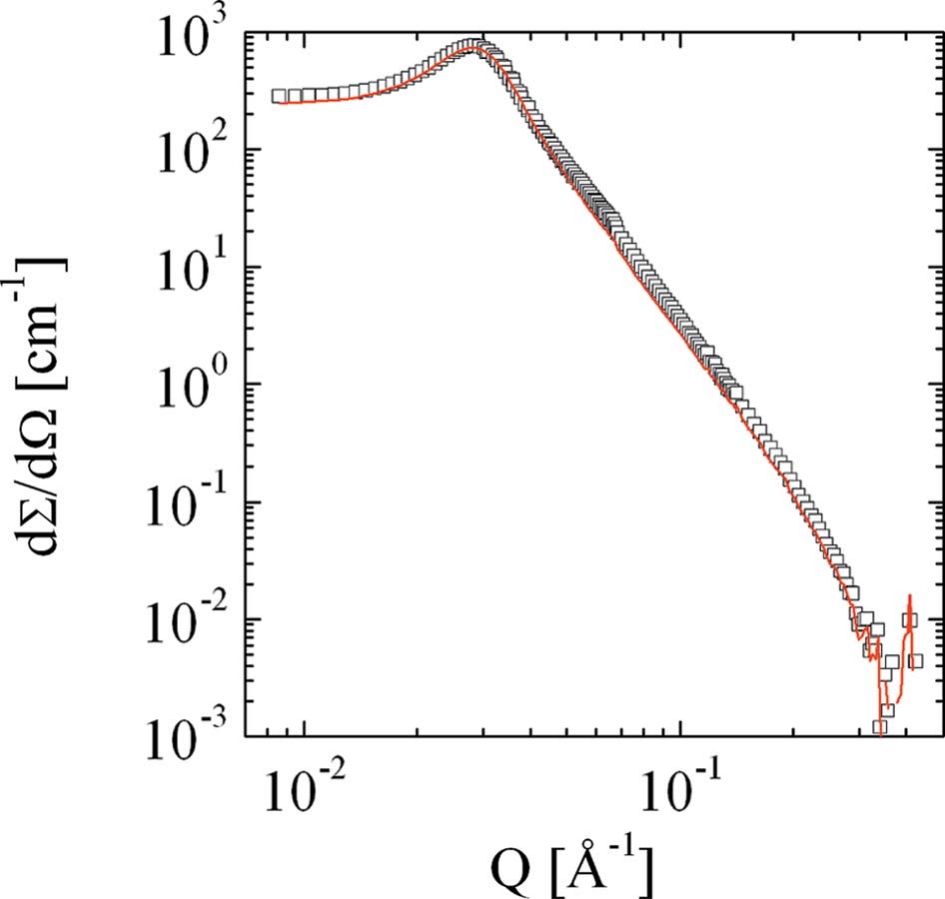
The macroscopic differential cross section as measured (empty squares) and deconvoluted by method 1 [equation (14)] (red line) (λ = 5 Å, *d* = 1 mm, *T* = 0.554).

**Figure 8:**
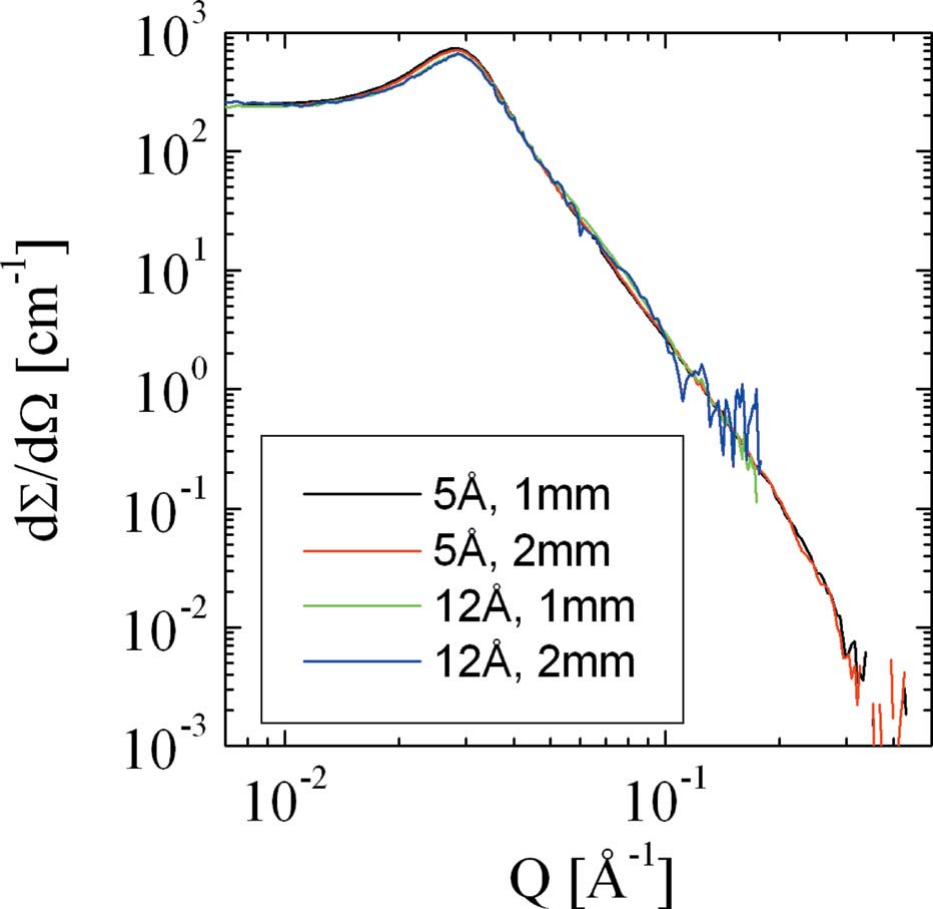
The macroscopic differential cross sections as deconvoluted with method 1 [equation (14)] from the original data shown in Fig. 6 ▸. The noise at the highest *Q* values is preserved.

We now discuss the multiple scattering correction of the data set for λ = 5 Å, *d* = 1 mm (Fig. 7 ▸), where we require only small corrections. Many data points are corrected towards slightly lower intensities, but the general shape of the curve is maintained. Note also that towards high *Q* the noise of the data set is preserved. Usually at these high *Q* values the statistical noise of the data becomes dominant and the high-*Q* cut-off no longer plays an important role. However, for theoretical calculations this may still be an issue, as seen below in Figs. 11 and 12. We now take this corrected data set as a reference for the larger corrections of the other data sets. These curves are presented in Fig. 8 ▸. In general, all of the important features are recovered (we had to assume a slightly higher background for the λ = 12 Å measurements owing to increased air scattering that was not properly subtracted by the empty-cell measurement, possibly due to a slightly erroneous transmission measurement). The most pronounced noise appears for the two measurements with the wavelength λ = 12 Å at high *Q*. Here, a substantial correction at *Q* = 0.06 Å^−1^ has taken place that has completely removed the pronounced shoulder. However, the correlation peak does not seem to be fully corrected for the λ = 12 Å, *d* = 2 mm measurement. On an absolute scale, the missing intensity is less than∼10%. Note that in the last case the transmission was only 3.1%.

**Figure 9:**
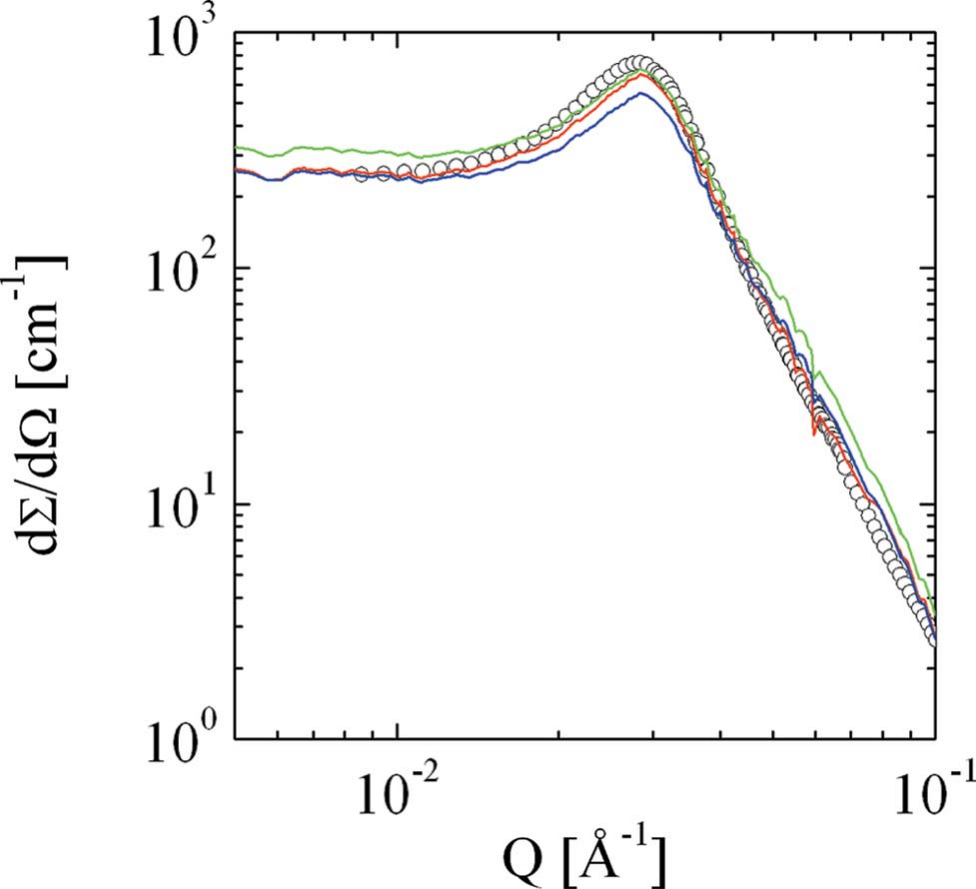
The deconvolutions by methods 1, 2 and 3 (red, blue and green lines, respectively) of the measurement (λ = 12 Å, *d* = 2 mm, *T* = 0.031) and the deconvolution with method 1 with weakest corrections (symbols, λ = 5 Å, *d* = 1 mm, *T* = 0.554). Methods 2 and 3 show the most substantial deviations from the ‘expected’ result (method 2 at the peak and method 3 at the forward scattering).

**Figure 10:**
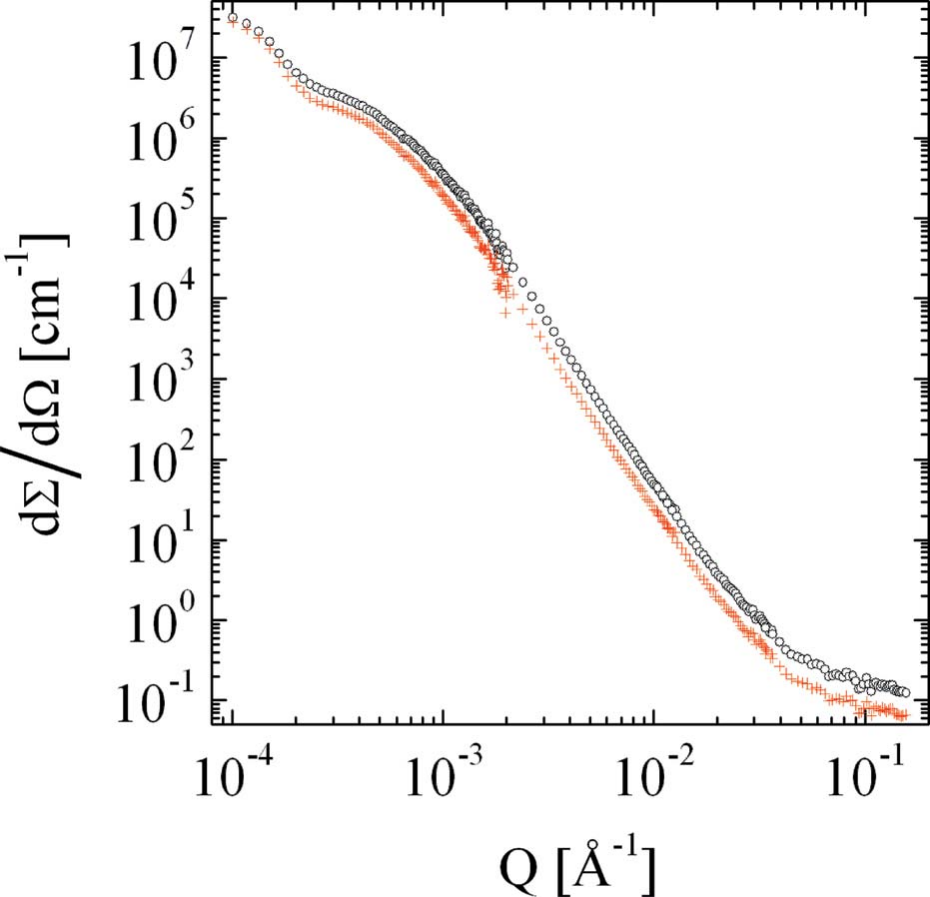
An example of small-angle scattering from the RO98 pHt membrane that is used in the desalination of potable water (Pipich *et al.*, 2020 ▸). The black circles indicate the calibrated apparent measurement including multiple scattering effects, and the red plus signs represent the data corrected by method 1. The intensities are reduced, but the Guinier scattering at the lowest *Q* values is preserved. The sample thickness was 300 µm and the transmission was *T* = 0.113.

We now focus on the desmearing methods (1–3). For the first data set (λ = 5 Å, *d* = 1 mm) the different results are almost indistinguishable. We therefore focus on the data requiring the largest corrections (Fig. 9 ▸). The differences between the three methods become obvious in the forward scattering and around the correlation peak. This demonstrates that method 1 is the most appropriate to correct data sets for multiple scattering, although it requires highly precise apparent absolute calibration to obtain the optimum results. Furthermore, we had to introduce a ‘fudge factor’ α = 0.057 cm for methods 2 and 3, which could not be reproduced exactly for other samples. One example from the literature (Pipich *et al.*, 2020 ▸) displays the scattering from a reverse osmosis membrane (Fig. 10 ▸). This measurement also included very small *Q* values down to 10^−4^ Å^−1^ using the VSANS instrument KWS3. The displayed correction is performed with method 1. Again, the curve is shifted to lower intensities and the statistical noise is treated well. The other methods did not agree well enough with method 1 and are not displayed here.

## 7. Examples for desmearing instrumental resolution

**Figure 11:**
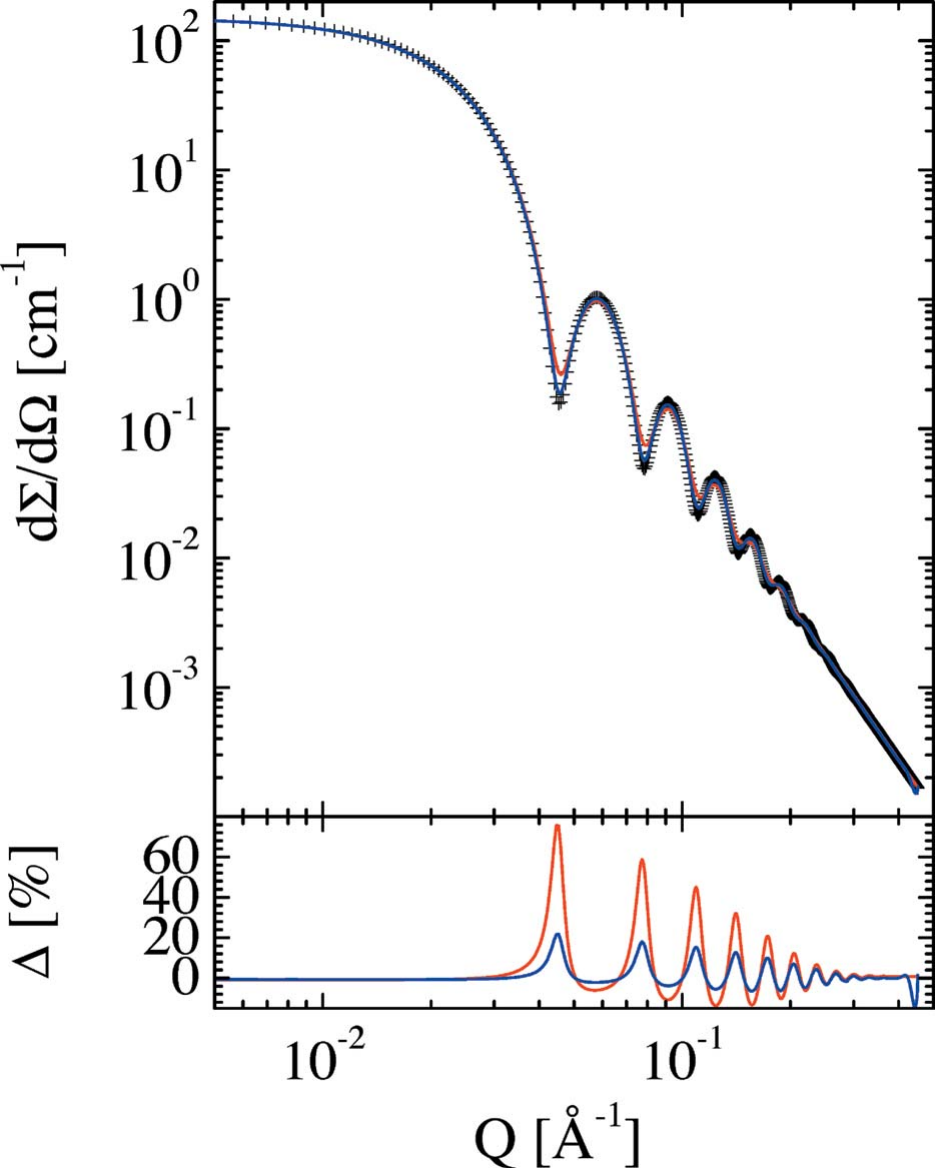
Simulated SANS curve for spherical particles of radius 100 Å with 5% polydispersity (standard deviation of a Gaussian distribution) but without instrumental smearing (symbols). The red curve displays the same simulated data including experimental smearing. The deconvolution of the instrumental smearing is depicted as the blue curve. The first simulation without instrumental smearing is recovered to within the error of the symbol size.

**Figure 12:**
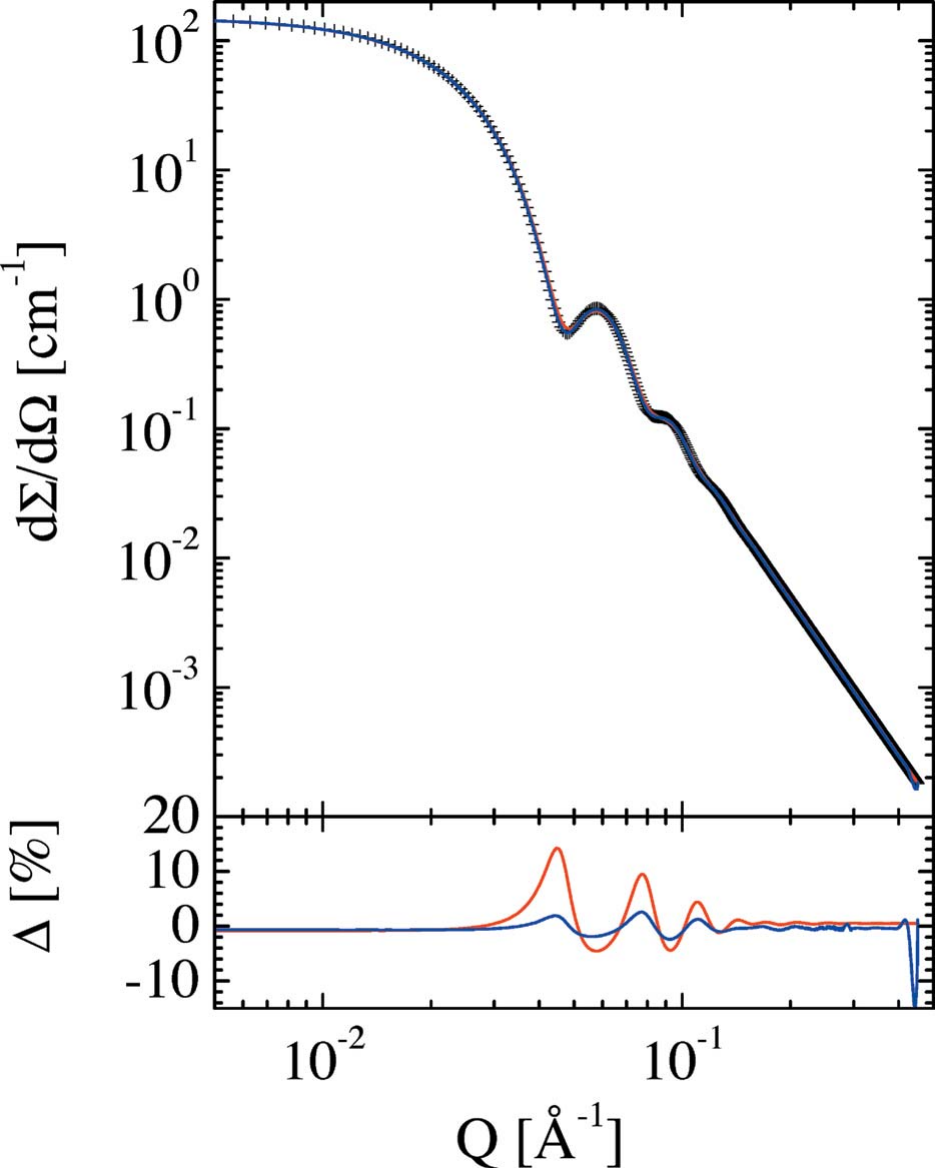
Simulated SANS curve for spherical particles of radius 100 Å with 10% polydispersity (standard deviation of a Gaussian distribution) but without instrumental smearing (symbols). The red curve displays the same simulated data including experimental smearing. The deconvolution of the instrumental smearing is depicted as the blue curve. The first simulation without instrumental smearing is recovered to within the error of the symbol size.

Finally, we discuss several examples for desmearing experimental resolution effects. For this, we smeared the theoretical scattering of spherical particles with different polydispersities in addition to the smearing applied by Pedersen *et al.* (1990 ▸). Examples are given in Figs. 11 ▸ and 12 ▸. The original scattering curves describe spherical particles with 100 Å radius and polydispersities of 5 and 10%. The red curves describe the simulation of experimental smearing using the Pedersen resolution function. The desmearing is displayed by the blue curves. Within the symbol size, the original scattering functions are recovered. The slight deviations from the original data points correspond to slightly higher polydispersities of 5.8 and 10.8%. Thus, the instrumental desmearing works very well to a very good approximation. The effect of the *Q* cut-off on the deviation, Δ (blue curve), is only noticeable at high *Q* values.

**Figure 13:**
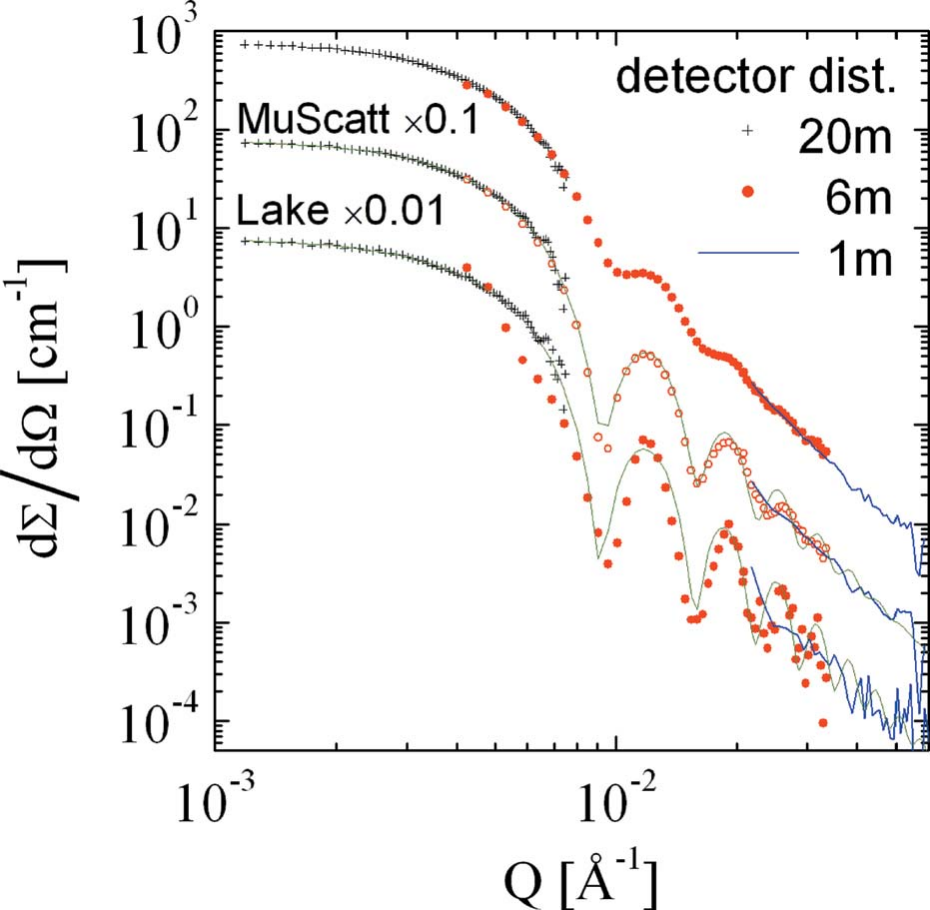
Experimental data from colloidal particles with a radius of 500 Å. The different symbols originate from different sample-to-detector distances of 20, 6 and 1 m. Each of the data sets was desmeared using the *MuScatt* routine and the Lake algorithm for slit desmearing (shifted down).

Another example, for experimental data, is displayed in Fig. 13 ▸. Here, we distinguish the different detector settings at 20, 6 and 1 m by different symbols. Each of the data sets was desmeared individually using the *MuScatt* (Frielinghaus, 2021 ▸) routine and the Lake (1967 ▸) algorithm in the *IRENA* software (Ilavsky & Jemian, 2009 ▸). The latter was developed for the rectangular slit smearing typically needed for USAXS and USANS. The noise of the 6 m data is strongly enhanced by the Lake algorithm towards higher *Q*. This is highly undesirable. In contrast, the *MuScatt* routines recover a scattering curve that can be well described by a polydisperse sphere (green curve). The triangular fringes and shifted minima in the Lake algorithm correction are a result of the erroneous assumption of slit desmearing – for SANS data a Gaussian desmearing would be more appropriate. Overall, *MuScatt* does not strongly exaggerate the fringes and correctly places the positions of the minima. Thus, we confirm the findings of Figs. 11 ▸ and 12 ▸. The overall results are therefore satisfactory and this approach may be applied more widely for reduced experimental data.

## 8. Recommended SANS data treatment

The general suggestion for reactor-based SANS data is that the instrumental desmearing be applied to different instrumental settings separately before the multiple scattering correction is performed. In this way, individual effects of different collimation settings are properly taken into account as needed for reactor-based instruments with a velocity selector. This instrumental resolution treatment can be performed with the routine deconv, which reads in four-column scattering data where the fourth column gives the standard deviation of the *Q* smearing, σ_*Q*_ (Å^−1^). The desmearing of the main routine in *MuScatt* may also be used for merged data sets of different detector distances. Here, one assumes that the entrance aperture smearing of the longest setting holds for all data and only the wavelength desmearing is applied correctly.

For time-of-flight SANS instruments, the different λ slices may be desmeared simultaneously in terms of resolution and multiple scattering with the *MuScatt* routine. Slices of 1 Å width seem to be well suited – at least for the suggested corrections. However, only after the different slices have been desmeared can they be combined to obtain a full-*Q*-range scattering function. A slim version of *MuScatt* uses method 1 for multiple scattering desmearing and reads four-column scattering data. A combination of the λ-dependent transmission and the λ slices may make the background subtraction more reliable.

In general, we propose the desmearing method 1 as the best choice for removing multiple scattering effects because it is most reliable and does not introduce a fudge factor. The current *MuScatt* routine is implemented in the current version of the SANS software *QtiSAS* (Pipich, 2021 ▸).

## 9. Summary

We have obtained population differential equations to describe the multiple scattering effects occurring at SANS instruments with different separable contributions: the coherent small-angle scattering, the elastic incoherent scattering and the inelastic incoherent scattering.

The inelastic scattering results from the thermalization of the neutrons. However, this thermalization can effectively be described by a single scattering event.

In this simplification, we assume additionally that it is a single wavelength to which the inelastic scattering evolves. The solutions of the differential equations agree well with the experimental data.

In practice, we still need to confirm the assumptions made in this article for routine measurement with solvents other than water, *i.e.* that the thermalization takes place by a single scattering event and results in a single wavelength. While it is hard to prove that the underlying theoretical concept would work in all practical examples, it could be tested by measuring H_2_O/D_2_O mixtures on a time-of-flight SANS instrument.

We have developed the program *MuScatt* (Frielinghaus, 2021 ▸), which deconvolutes both multiple scattering effects and instrumental resolution. The algorithms have been optimized such that the instrumental noise is preserved well and not amplified. This fidelity to the data allows us first to correct the data at a good noise level and subsequently to describe the data by other models that can then be used to obtain the statistical errors of the model fitting at a representative level.

## Appendix A List of symbols

*z*: dimension along the axis of the collimated neutrons.

*x*, *y*: dimensions in the detector plane.

*Q*: modulus of the scattering vector.

**Q** = (*Q*_*x*_, *Q*_*y*_): vectorial *Q* in detector plane.

**r** = (*x*, *y*): vector in real space in detector plane.

*a*(**Q**): function in *Q* space.

: same function in *r* space. The formalism of multiple scattering demands these two-dimensional Fourier transforms.

ϑ: scattering angle.

ϕ: polar angle.

λ: wavelength of incoming neutrons.

λ_2_: wavelength after inelastic scattering.

: incident intensity at the sample.

*I*_0_: primary intensity.

*i*_1_: coherently scattered intensity.

*I*_1_: integral coherently scattered intensity  .

*j*_+_: incoherent elastic scattering in transmission.

*j*_−_: incoherent elastic back-scattering.

*k*_+_: incoherent inelastic scattering in transmission.

*k*_−_: incoherent inelastic back-scattering.

: integral over the solid angle in transmission or back-scattering hemisphere  ,  .

: integral over the solid angle with respect to **Q**′,  =  =  .

Σ_c_: cross section of coherent scattering,  .

: macroscopic differential cross section of coherent scattering.

: apparent macroscopic differential cross section of an experiment, *i.e.*  .

Σ_i_: cross section of elastic incoherent scattering.

Σ_a_: cross section of absorption.

Σ_ii_: cross section of inelastic incoherent scattering.

Σ_i2_: cross section of elastic incoherent scattering at λ_2_.

Σ_a2_: cross section of absorption at λ_2_.

Σ_t_: total cross section Σ_c_ + Σ_i_ + Σ_ii_ + Σ_a_.

Σ_iia_ = Σ_i_ + Σ_ii_ + Σ_a_.

Σ_ia2_ = Σ_i2_ + Σ_a2_.

*T*_t_: total transmission, *i.e.*  .

*T*: experimental transmission, for us *T* = *T*_t_.

*T*_iia_: partial transmission, *i.e.*  .

*F*: Chandrasekhar’s scattering function in transmission.

*B*: Chandrasekhar’s back-scattering function.

*X*, *Y*: Chandrasekhar’s *X* and *Y* functions.

τ_1_: optical thickness Σ_iia_ *d*.

μ: cosine of exit angle, *i.e.*  .

μ_0_: cosine of incident angle, *i.e.*  .

ϖ_0_: albedo, for elastic incoherent scattering Σ_i_/Σ_iia_.

*C*_0_, *C*_1_, *C*_2_: coefficients for inner elastic incoherent intensity.

Σ_1_, Σ_2_: effective cross sections for that description.

*k*, *k*_0_: wavevectors, *i.e.* 2π/λ_2_, 2π/λ.
